# S/MAR sequence confers long-term mitotic stability on non-integrating lentiviral vector episomes without selection

**DOI:** 10.1093/nar/gku082

**Published:** 2014-01-27

**Authors:** Santhosh Chakkaramakkil Verghese, Natalya A. Goloviznina, Amy M. Skinner, Hans J. Lipps, Peter Kurre

**Affiliations:** ^1^Department of Pediatrics, Papé Family Pediatric Research Institute, Oregon Health & Science University, Portland, OR 97239, USA, ^2^Department of Surgery/Surgical Oncology, Oregon Health & Science University, Portland, OR 97239, USA, ^3^Center for Biomedical Education and Research, Institute of Cell Biology, University of Witten/Herdecke, Witten 58453, Germany, ^4^Oregon Stem Cell Center, Oregon Health & Science University, Portland, OR 97239, USA and ^5^Department of Cell & Developmental Biology Oregon Health & Science University, Portland, OR 97239, USA

## Abstract

Insertional oncogene activation and aberrant splicing have proved to be major setbacks for retroviral stem cell gene therapy. Integrase-deficient human immunodeficiency virus-1-derived vectors provide a potentially safer approach, but their circular genomes are rapidly lost during cell division. Here we describe a novel lentiviral vector (LV) that incorporates human ß-interferon scaffold/matrix-associated region sequences to provide an origin of replication for long-term mitotic maintenance of the episomal LTR circles. The resulting ‘anchoring’ non-integrating lentiviral vector (aniLV) achieved initial transduction rates comparable with integrating vector followed by progressive establishment of long-term episomal expression in a subset of cells. Analysis of aniLV-transduced single cell-derived clones maintained without selective pressure for >100 rounds of cell division showed sustained transgene expression from episomes and provided molecular evidence for long-term episome maintenance. To evaluate aniLV performance in primary cells, we transduced lineage-depleted murine hematopoietic progenitor cells, observing GFP expression in clonogenic progenitor colonies and peripheral blood leukocyte chimerism following transplantation into conditioned hosts. In aggregate, our studies suggest that scaffold/matrix-associated region elements can serve as molecular anchors for non-integrating lentivector episomes, providing sustained gene expression through successive rounds of cell division and progenitor differentiation *in vitro* and *in vivo*.

## INTRODUCTION

Retroviral vectors provide maneuverable targeted tropism and stable genomic integration, ideally suited for stem cell gene therapy. Human immunodeficiency virus-1 (HIV-1)-derived lentiviral vectors (LVs) provide specific additional advantages for efficient gene delivery via the transport of the vector genomes into the nucleus of both dividing and non-dividing cells (1). Although a desired feature for heritable genetic modification of stem cells, vector integration can induce insertional oncogene activation, alternative splicing, aberrant transcripts and read-through transcription of endogenous genes from cellular genomic DNA (2,3). For some non-dividing tissue targets, use of non-integrating lentiviral vector (niLV) and non-viral vector provides a potentially safer approach that drastically reduces integration by several orders of magnitude and minimizes the likelihood of gaining replication competence (4–8). However, the niLV vector genomes are rapidly lost during cell division with a concomitant decrease in transgene expression that precludes their use for hematopoietic stem cell gene therapy (9). Non-integration is an advantage for safety in stem cell gene therapy only if there is stable and long-term maintenance of the vector genome within the nucleus of replicating cells.

During the lentiviral life cycle and on cell entry of pseudotyped vectors derived from lentiviruses, the linear DNA products that serve proviral integration as well as the circular DNA species arising from recombination events involving the viral LTR (LTR circles) are formed within the host cell (10,11). These LTR circles contain no origin of replication to propagate them during cell division. In certain viral vectors, host cell-dependent DNA replication is accomplished by viral sequences that facilitate nuclear retention and replication. The incorporation of heterologous virus-derived elements from Epstein–Barr virus or Simian virus (SV40) in non-integrating viral vectors can provide episomal persistence, but also heighten the potential risk of transformation or vector immunogenicity (12–14).

The mammalian genome harbors elements that interact with the nuclear matrix to generate chromosome domains, effectively position the DNA within the nuclear matrix and modulate DNA replication during cell division (15–17). Scaffold/matrix-associated regions (S/MARs) DNA elements linked to an expression unit can recruit non-viral (i.e. cellular) factors to promote episomal replication and mitotic stability (18–20). Several viral and non-viral vectors have been developed using S/MAR sequence derived from the human β-interferon locus to harness these properties (21–26). However, existing studies using non-viral S/MAR vectors and most hybrid viral vectors provide comparatively low gene transfer efficiency to hematopoietic progenitor cells (HPC) (22,27). Additionally, these hybrid vectors lack the capacity for nuclear translocation of the vector genome and require breakdown of the nuclear membrane during mitosis for episome establishment (28).

In this study, we engineered human ß-interferon gene cluster-derived S/MAR sequence in a non-integrating third-generation lentiviral transfer vector. The resulting anchoring non-integrating lentiviral vector (termed ‘aniLV’) ‘anchored’ the transgene expression cassette to the cellular genome and was retained in clonally derived dividing cells. In studies targeting murine HPC for transduction, we observed gene expression in progenitor colonies and *in vivo*. Our proof-of-concept studies establish the mitotically stable retention of lentivector episomes, allowing gene delivery to HPCs that takes full advantage of lentivector tropism and nuclear import.

## MATERIALS AND METHODS

### Plasmids and cloning

The third-generation HIV-1-based transfer plasmid pLVCG was used to incorporate the 2-kb S/MAR fragment (a kind gift from Dr Francesco Galimi). In the four-plasmid vector system used in this study, pLVCG was used with the helper plasmids pLP1 (coding HIV-1 Gag-Pol), pLP2 (coding HIV-1 Rev) and pLP/VSVG (encoding vesicular stomatitis viral envelope glycoprotein) (GIBCO-Invitrogen) to produce the vector particles. The (D64V) integrase mutant HIV-1 Gag-Pol plasmid, pCD/NL2 ΔInt (a kind gift from Dr Helmut Hanenberg) was used, instead of pLP1, to produce niLV stock (10,29). Human ß-interferon gene cluster-derived S/MAR sequence and GFP were released from pEpi plasmid (25) after digest with EcoRI and NheI. pLVCG was digested with EcoRI and XbaI and ligated with GFP-S/MAR fragment. The resulting plasmid was designated pLV-S/MAR and used to produce iLV-S/MAR and aniLV vector particles.

### Cell culture

HEK 293T human kidney fibroblasts, HS5 cells and murine NIH3T3 cells were grown in Dulbecco’s modified Eagle’s medium (DMEM) supplemented with 10% fetal bovine serum (FBS) and 1% Pen/Strep. HPCs were cultured in Iscove’s modified Dulbecco’s medium containing 10% FBS, 10% horse serum and 1% penicillin/streptomycin, as previously described (30).

### Vector production

Human embryonic kidney 293T cells were seeded at a density of 1.5 × 10^7^ cells per 15-cm tissue culture dish (Corning, NY, USA) and precoated with 0.01% poly-l-lysine (Sigma, MO, USA). The integrating viral vector stocks were produced by using the helper constructs pLP1, pLP2 and pLP/VSVG (31). The non-integrating viral vector stocks were produced by using the helper constructs pCD/NL2 ΔInt, pLP2 and pLP/VSVG plasmids. The lentiviral transfer vector plasmids pLVCG and pLV-S/MAR were co-transfected with the helper constructs to produce integrating and non-integrating vector stocks. As previously described (30), calcium phosphate transfection was performed in the presence of DMEM (Gibco), 10% FBS (Gibco) and 1% penicillin/streptomycin (Pen/Strep, Gibco). Vector supernatant was harvested 36, 48 and 72 h later, filtered through a 0.45-µm filter, pooled and ultra-concentrated over 30 h at 7300 RCF, and the pellet was resuspended in Iscove’s media (Gibco) and stored at −86°C until use. Limiting dilution titers were determined by FACS and calculated using 293T cells, as previously described (31). For transduction, cells were washed and resuspended in corresponding media (described below), with 8 µg/ml protamine sulfate (MP Biomedicals). Transductions took place overnight at 37°C followed by wash and culture in DMEM.

### Animal husbandry

C57BL/6 mice were group-housed and allowed *ad libitum* access to standard chow pellets (Purina Laboratory Rodent Diet 5001; Ralston Purina Co., MO, USA). Whole bone marrow cells were collected by flushing femurs and tibias from 8- to 12-week-old mice (CD45.1/2) with Iscove’s modified Dulbecco’s media. Samples were depleted of red cells by hemolysis and lineage-depleted using an Easy Sep Mouse Hematopoietic Progenitor Cell Enrichment kit according to manufacturer’s instructions (StemCell Technologies Inc., Vancouver, Canada). Following LV transduction at multiplicity of infection (MOI) 10 in the presence of 8 µg/ml protamine sulfate, cells were incubated overnight and washed twice in phosphate-buffered saline (PBS), resuspended in 200 μl Hank’s balanced salt solution and injected intravenously into myeloablated (750 cGy) recipients. Following transplantation, retro-orbital eye bleeds were performed at intervals, and white blood cells were analyzed for transgene expression by flow cytometry (30). All animal studies were approved by the OHSU institutional animal care and use committee.

### Flow cytometry

GFP expression was analyzed with a FACS-Calibur instrument (BD Biosciences) and processed using Flow Jo software (Tree Star, Ashland, OR, USA). At least 10 000 events were collected for any given experiment. Mean fluorescence intensities (MFI) were also analyzed using FlowJo software. The software determines the test result by comparing the MFI of individual cells against total population of GFP-positive cells. For stringent clonal selection, GFP-positive cells were sorted by InFlux Cell Sorter (BD Biosciences). Sorted cells were washed twice in DMEM and propagated in 10-cm plates at 100 cells per plate. After 2 weeks, single colonies were selected, split and propagated further in separate flasks.

### Colony rescue assay

To exclude bacterial plasmid contamination, chemically competent Dh5α *Escherichia coli* cells were transformed with S/MAR-containing plasmids pEpi and pLV-S/MAR and non-S/MAR plasmids pLVCG and pGFP-Vpr by using 10 ng of plasmid DNA. In all, 500 ng of whole genomic DNA from aniLV- and iLV-transduced cells were used in transformation. Transformed bacterial cells were plated on ampicillin-containing agar plates and incubated at 37°C overnight. The colonies were counted and analyzed for plasmid DNA.

### HIRT DNA extraction

Cells grown in 15-cm plates were harvested after trypsin treatment and washed with PBS. Cell pellets were suspended in 200 μl PBS supplemented with 2.4 ml of solution A [0.2 N NaOH, 1% sodium dodecyl sulphate (SDS)]. The solution was thoroughly mixed until clear, and 1.2 ml of solution B was added (5 M potassium acetate, 11.5% glacial acetic acid) and mixed by inverting, followed by incubation on ice for 10 min. The precipitate was pelleted by centrifuging at 12 000 rpm for 30 min. Supernatant was carefully collected and transferred to a fresh tube. DNA was precipitated by adding 0.6 volume of 100% ethanol and incubated in −80°C for 30 min followed by centrifugation at 12 000 rpm for 30 min. Supernatant was discarded after the spin, and the pellet was washed with 70% ethanol. Supernatant was discarded, and the tubes were air-dried for 15 min. The pellet was solubilized by adding 500 μl of TE and incubated at 56°C overnight after adding 50 μg of Proteinase K and 1 U of RNase A. DNA was further purified by Qiaquick Spin column (Qiagen, Hilden, Germany) according to the manufacturer’s protocol.

### Polymerase Chain Reaction

Polymerase chain reaction (PCR) was used to analyze the LV-transduced cellular and HIRT DNA. Twenty-five microliters of reactions were set up by adding DNA template followed by 1× Taq PCR buffer, 200 μM dNTPs, 0.2 μM primers and 1.25 U of Taq polymerase (Invitrogen). Primer sequences used in the PCR reactions are polypurine tract-F (PPT-F; 5′-acaaggcagctgtagatcttagccac-3′), primer binding site-R (PBS-R; 5′-ctttcgctttcaggtccctgttcg-3′) and GFP R (5′-ttcaccggggtgtgcccatcctg-3′). Thermocycler conditions: denaturation at 95°C for 5 min, followed by 35 cycles denaturation (95°C–1 min), annealing (65°C–1 min) and elongation (72°C–5 min). Terminal elongation at 72°C for 10 min was performed followed by hold temperature at 12°C. Primer sequences used in the Alu-PCR reactions are PPT-F and Alu-1 (5′-tcccagctactggggaggctgagg-3′. Thermocycler conditions: denaturation at 95°C for 5 min, followed by 35 cycles denaturation (95°C–1 min), annealing (50°C–30 s) and elongation (72°C–1.5 min). Terminal elongation at 72°C for 10 min was performed followed by hold temperature at 12°C. Reactions were analyzed by agarose gel electrophoresis.

### Quantitative real-time PCR

DNA was extracted from vector-transduced 293T cells using DNeasy Blood and Tissue kit (Qiagen) and quantified using Nanodrop 2000c (Thermo). For quantitative PCR (qPCR), GFP primers, GFP F (5′-caagggcgaggagctgttgacc-3′) and GFP R (5′-tgtggcggatcttgaagttcacc-3′) were used. Primer sequences used to amplify and quantitate 2-LTR sequences are RU5 F (5′-ggctaactagggaacccactgcttaag-3′) and RU5 R (5′-agctcccaggtcagatctggtc-3′). Relative quantification was calculated using ΔΔ*C*_T_ algorithm, and the copy number was calculated based on GFP copy number standard.

### DNA sequencing

LV DNA was sequenced by Applied Biosystem’s 16-capillary 3130xl automated sequence analysis system by the OHSU sequencing core. The DNA fraction of the reaction mixture contains 6.4 pmol of primer (PPT-F, PBS-R) and 30 ng of purified DNA. Sequences were analyzed by Sequencher and BLAST software (NCBI).

### Southern blot experiments

Genomic DNA was isolated by using the DNeasy Blood and Tissue kit (Qiagen). For Southern hybridization, 10 µg of genomic DNA was used, subjected to electrophoresis in agarose gels and transferred to a positively charged nylon membrane (Roche, Basel CH) according to manufacturer instructions. The hybridized DNA was detected according to the instructions of the manufacturer (Roche) by using the digoxigenin (DIG) non-radioactive nucleic acid labeling and detection system. DNA was labeled with DIG-dUTP by using Klenow fragment, and the DIG-labeled DNAs were used as probes for experiments after they were made single stranded by boiling for 10 min, followed by chilling in ice. Blots were incubated with the labeled probes for 16 h at 65°C in hybridization solution (Roche). The membranes were prewashed twice at room temperature with 2 × SSC (1× SSC is 0.15 M NaCl plus 0.015 M sodium citrate) containing 1% (wt/vol) SDS for 5 min, and this was followed by two stringency washes with 0.1× SSC–0.1% SDS for 15 min at 65°C. Color detection of the hybridized probes was carried out by using the instructions of the manufacturer (Roche) and NBT/BCIP as the detection reagent.

### Florescent *in situ* hybridization experiments

Transduced and selected aniLV/293T cell clones were used for the florescent *in situ* hybridization (FISH) experiment. In all, 10^6^ cells were arrested in mitosis by incubating them with 0.1 μg/ml colcemid for 16 h at 37°C. The cells were carefully resuspended in 2 ml of 75 mM KCl solution for 20 min at room temperature, then the cells were fixed overnight using ice-cold methanol:glacial acetic acid (3:1) at −20°C and collected again by centrifugation at 4°C. Metaphase spreads were prepared by dropping 20 µl of fixed cells onto HCl-etched 22 × 22 mm coverslips and incubated at 55°C in a humid chamber. The metaphase spreads were aged overnight at room temperature before beginning FISH. After aging, samples were digested with 200 mg/ml RNase A for 30 min at 37°C, then washed three times in 1× PBS and placed in 50% formamide in 2× SSC for 2–4 days at 4°C. FISH was performed as previously reported (16,25,32).

### Statistics

Statistical significance was determined by performing a paired two-tailed Student’s *t* test. Individual figure panels indicate whether equal or unequal variance was assumed. *P*-values of <0.05 were considered statistically significant.

## RESULTS

### Effect of S/MAR sequence on transgene expression from the lentiviral backbone

**Figure 1.**
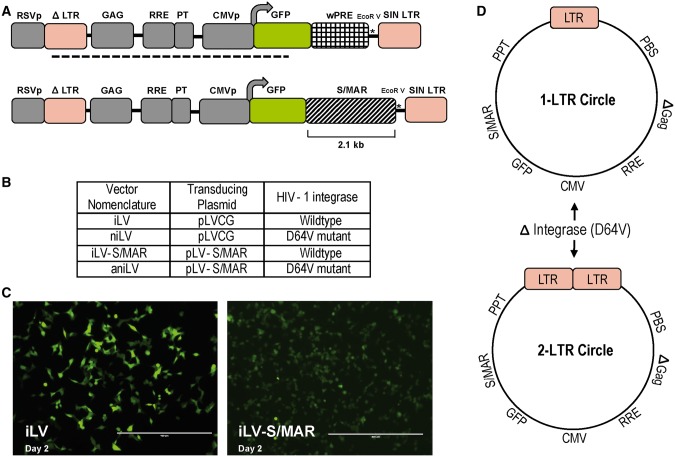
Effect of wPRE replacement with S/MAR sequence on transgene expression and production of non-integrating vector episomes. (**A**) Diagram of HIV-1-based self-inactivating LV pLVCG with wPRE cloned within the transgene expression cassette pLVCG harboring human S/MAR from the ß-interferon gene cluster. The GFP-wPRE fragment was released by digestion with XbaI and EcoRI to replace with the S/MAR fragment. The dotted line corresponds to the DIG-labeled probe binding site. S/MAR is cloned at the transcriptionally active region between GFP reporter ORF and 3′LTR after replacing the wPRE fragment in LVCG plasmid. (**B**) Four LVs classified on integration status and S/MAR presence were used in the study. iLV: integrating lentiviral vector harboring wPRE sequence, niLV: non-integrating lentiviral vector harboring wPRE, iLV-S/MAR: integrating lentiviral vector harboring S/MAR sequence, aniLV: anchoring non-integrating LV harboring S/MAR sequence. (**C**) Microscopy images of GFP expression from iLV and iLV-S/MAR-transduced 293T cells at the day-2 time point. Integrating vector stocks were produced by using the helper plasmids pLP-1, pLP-2 and pLP/VSVG. Non-integrating vector stocks were produced by using the helper plasmids pCD/NL2 ΔInt, pLP-2 and pLP/VSVG. (**D**) Diagram representing the LTR circle formation due to abortive integration of niLVs. The class I integrase mutant used to produce niLVs is a D64V mutant that limits IN function regarding cleavage and integration but maintains normal levels of viral DNA. The niLVs are produced by using the helper plasmids pCD/NL2 ΔInt, pLP-2 and pLP/VSVG. After transduction, the abortive integration event of the vector genome results the formation of reverse-transcribed circular DNA vector genomes with one or two LTRs. All LTR sequences used in this study are promoterless self-inactivating (SIN) vector sequences.

The 2-kb S/MAR sequence from the human ß-interferon gene cluster was derived from pEpi plasmid and cloned between GFP reporter ORF and the 3′LTR of the plasmid pLVCG (Figure 1A). In the process, the woodchuck hepatitis virus post-transcriptional regulatory element (wPRE fragment), generally used as a transcriptional enhancer of gene expression, was replaced by the S/MAR fragment in the pLVCG plasmid (33). The replacement of virus-derived wPRE with cell-derived S/MAR fragment was intended to reduce the viral elements in the vector design while reducing the size of the vector genome to provide sufficient transgene cloning capacity. Additionally, this design allowed analysis of the S/MAR-mediated transgene expression without the added variable of wPRE as a transcriptional enhancer. The LVs used in this study were classified based on vector integration status and S/MAR-containing transducing plasmids used during vector production. We used the class I integrase-defective packaging plasmid pCD/NL2 ΔInt (D64V), to produce niLV stocks (Figure 1B). From a biosafety point of view, this design maximizes the use of human sequences and avoids heterologous viral sequences for ‘anchoring’ non-integrating vector episomes in dividing cells. The replacement of the viral transcriptional enhancer with the cellular regulatory element S/MAR resulted in lower GFP expression in iLV-S/MAR-transduced cells compared with iLV (harboring wPRE)-transduced cells (Figure 1C). The data illustrate that the comparatively lower gene expression from episomes (below) may in part be due to elimination of the wPRE sequence. For a direct comparison, all versions of the vector were produced by standard protocol (30) in 293T cells and maintained in culture without selection. In the presence of integration defective Gag-Pol helper protein, linear vector DNA predominantly forms non-integrating 1- and 2-LTR circles at a 9:1 ratio (34,35) (Figure 1D).

### Establishment of aniLV episomes and transgene expression

**Figure 2.**
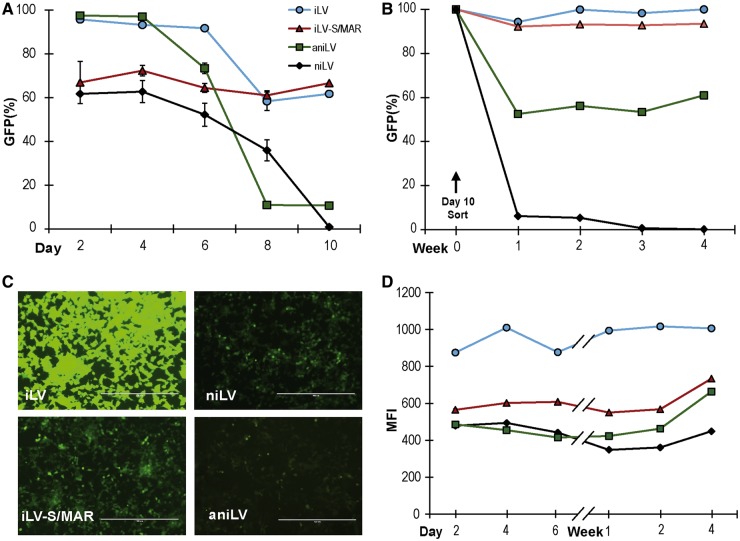
Transgene expression from LVs and long-term GFP expression from aniLV-transduced 293T cells. (**A**) Time-course evaluation of GFP expression from the LV-transduced 293T cells at post-transduction time points. The population of GFP-positive cells was analyzed at intervals to measure the effect of wPRE replacement by S/MAR and functional integrase replacement by defective integrase. (**B**) Long-term stability of GFP expression from the aniLV-transduced and enriched cells after sorting. Mitotic stability and rate of gene expression from integration defective S/MAR containing LV in relation to control vectors. (**C**) Microscopy images of GFP florescence from the LV-transduced 293T cells. GFP signal was consistently lower in integrase-defective niLV vectors and S/MAR harboring vectors. Scale bars: 400 µm. (**D**) Comparison of GFP expression levels (MFI) from LV-transduced 293T cells two days post-transduction. GFP expression was significantly lower in integrase-defective wPRE-harboring vector than the integrating wPRE vector.

Following LV transduction of 293T cells with integrating and niLV, containing wPRE or S/MAR sequences, respectively, GFP frequency in the bulk population (i.e. transduction efficiency in dividing cells) was serially tracked for 10 days. (Figure 2A). Data illustrate transduction frequency at the earliest time point with a declining proportion of GFP-positive cells after transduction with niLVs, for both aniLV (S/MAR^+^) and niLV (no S/MAR). Establishment rates of aniLV vector-derived gene expression were analyzed in additional human and murine cell lines (HS5 and NIH3T3 cells, respectively), and we observed a similar initial loss of gene expression followed by stabilization of GFP-positive population in aniLV-transduced cells (Supplementary Figure S1A, B). The respective stock vector titers to calculate the MOI used in the experiments were calculated by FACS analysis and verified by Q-PCR. (Supplementary Figure S1C). Next, we flow cytometrically sorted GFP-expressing events from all four transduction experiments in 293T cells to purity (100%) and followed GFP frequency and MFI in these cultures. Unlike the niLV control population that predictably lost expressing events, the GFP-positive population in aniLV-transduced cells stabilized after ∼10 cell divisions (Figure 2B). Circular episomes from niLVs generally provide lower levels of transgene expression, i.e. GFP MFI, compared with integrated vector genomes, presumably because iLV integration into transcriptionally active regions of the chromatin provides improved access to transcription factors compared with niLVs (36). Accordingly, transgene expression (i.e. MFI) in cells with established episomes was stable, if consistently lower in aniLV- and niLV-transduced cells compared with iLV- and iLV-S/MAR-transduced cells, resulting in an ∼30–50% reduction in MFI from aniLV compared with the integrating counterparts (Figure 2C). It is important to remember that the MFI measures how bright the GFP signal is within the GFP-positive gate, irrespective of the number of contributing events. It is expected that even a small number of residual GFP events in the case of the niLV vector genome will therefore register a measurable MFI (Figure 2D).

### Long-term aniLV performance in dividing cells

**Figure 3.**
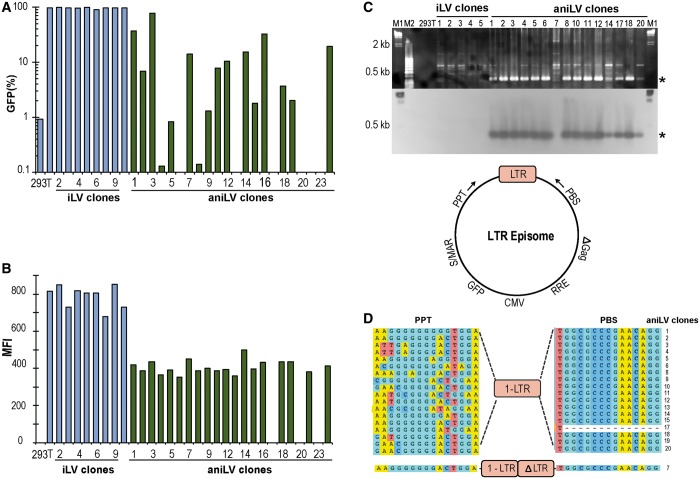
Episomal persistence and gene expression in 293T LV clones. (**A**) GFP-positive cells in LV-transduced single cell clones of long-term cultured 293T cells. All clones were maintained in culture without selection pressure. Clonal variability of GFP-positive cells in aniLV clones (green) was observed compared with iLV-transduced 293T clones (blue). (**B**) MFI in LV-transduced single cell clones 8 weeks after transduction with aniLV or iLV vector at matched MOI. Decreased MFI from aniLV clones compared with iLV clones. (**C**). Agarose gel electrophoresis of PCR and Southern blot on LTR junctions (bottom) in aniLV clones using PPT F and PBS R primers. M1: DIG-labeled Lambda HindIII DNA ladder (Roche), M2: 50 bp DNA ladder. Numbers represent DNA template from the respective clone used for PCR and Southern blot analysis. (**D**) Sequence of LTR circles of aniLV clones and the primer orientation on the vector episome. LTR junctions of the episomes are sequenced and analyzed. Complete 2-LTR episomes are not observed in the screened clones. Eighteen clones have 1-LTR junctions and one clone showed one full-length LTR and a partial LTR (ΔLTR) spanning by PPT and PBS sequence on both sites.

For detailed long-term studies of aniLV performance, single cell clones were generated from the bulk-sorted population of GFP-positive 293T cells. These clones were expanded and have since been in culture for >100 cell divisions. Each clone, representing progeny from a single transduced cell, was maintained without selection pressure to analyze the retention potential of the S/MAR harboring vectors. Gene expression from niLVs is generally lower than iLV, but S/MAR episomes are additionally subject to host cell chromatin modifications (34,37). Not surprisingly then, the aniLV clones showed a wide variability in the proportion of GFP-positive cells compared with iLV clones (Figure 3A). Intriguingly, the fluorescence intensity (MFI) in aniLV/293T clones was stable, albeit generally lower than from iLV clones (Figure 3B). To analyze the variability of GFP expression from the aniLV clones, three of the aniLV clones were further sorted for GFP-positive and GFP-negative cells. On expansion of these cells, GFP expression was analyzed by FACS followed by the detection of GFP cassette and vector episomes by PCR. (Supplementary Figure S2A–C). The low-level (<5%) GFP expression in cells sorted in the ‘GFP-negative gate’ may simply reflect incomplete exclusion of expressing event in the sort gate. However, the presence of prominent LTR and GFP–PCR signals in both populations certainly suggests variable transgene expression and is consistent with potential silencing of episomes in subclones by a mechanism not yet known.

The aniLV/293T clones also provided an opportunity to formally confirm the presence of episomal DNA. We extracted cellular DNA from the clones and performed PCR to determine the presence of vector-derived LTR circles using episome-specific primers to the 5′ flanking PPT and 3′ PBS. By definition these primers will not amplify linear sequence, including proviral (i.e. genomic) integrants. For added stringency, LTR junction-specific PCR was followed by Southern hybridization using an LTR-specific hybridization probe confirming the presence of LTR episomes in aniLV-transduced, actively dividing 293T cell clones without selection (Figure 3C)*.* For further validation, we also sequenced the PCR amplicons, demonstrating the predominance of 1-LTR circles in aniLV/293T clones (Figure 3D). Additionally, we observed that mutations clustered in the PPT region of the sequenced clones compared with the PBS region that spans the amplified LTR circle sequence.

Once packaged, the aniLV vector used here contains neither a mammalian, nor a bacterial antibiotic selection cassette. To specifically exclude S/MAR-containing (transfection) plasmid contamination as a source of GFP expression in the aniLV-transduced cells, we performed bacterial colony rescue assays (Supplementary Figure S3A, B). DNA was extracted from the vector-transduced and plasmid-transfected (control) cells, transformed into competent *E. coli* cells and analyzed for colony formation. The absence of bacterial colonies from DNA of LV-transduced cells argues against the possible plasmid contamination during vector preparation. Detection and quantification of vector genome-derived 1-LTR and 2-LTR episomes was performed to further negate the presence of plasmid DNA in the transduced cells and to verify the LTR junctions in the aniLV-transduced and unsorted 293T cells. 1-LTR and 2-LTR DNA amplified from 293T cells at several time points from day 1 post-transduction up to 5 weeks further supports the presence of vector episomes in these unsorted cells without antibiotic selection (Supplementary Figure S3C). The higher prevalence of 1-LTR DNA was observed similar to aniLV-293T clones.

### Episome persistence in aniLV-transduced clones

**Figure 4.**
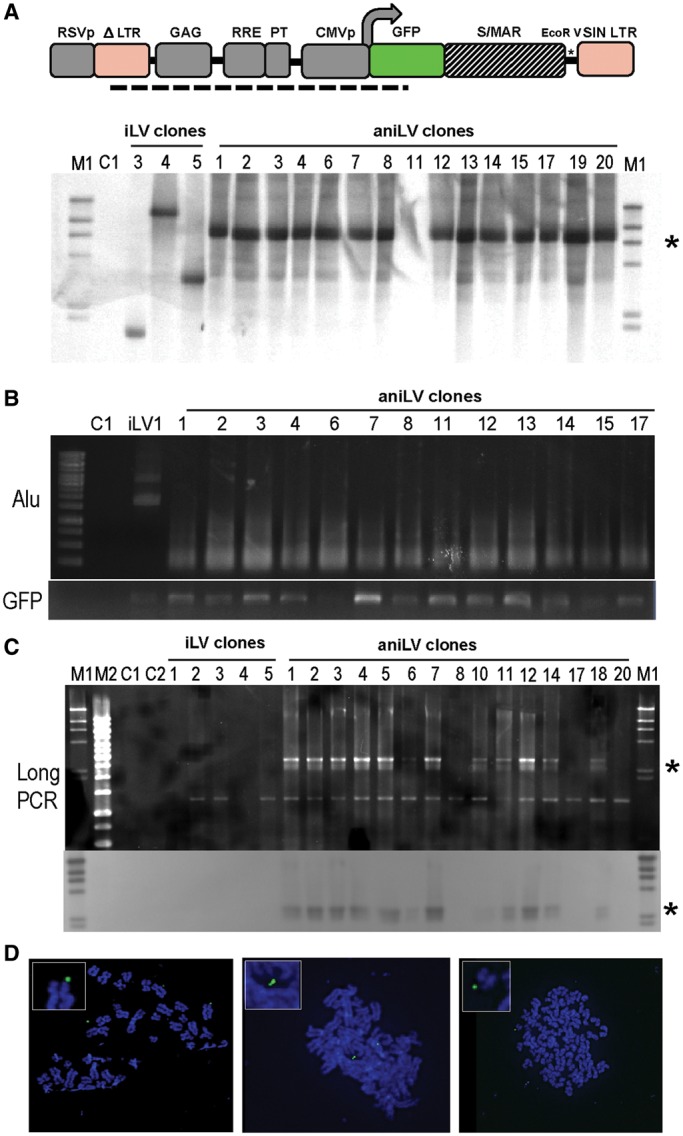
Mitotic stability and episomal status of anchoring niLV. (**A**) Southern blot analysis to identify the episomal and integrated vector copies. An EcoRV site located upstream to GFP ORF was used to linearize the vector. EcoRV-digested DNA samples were used, and the signal was detected by DIG-labeled probe as shown in Figure 1A. A single ≈6-kb DNA signal was observed in the analyzed aniLV clones. No signal was observed to indicate integrated copies in the analyzed aniLV clones. M1: DIG-labeled Lambda HindIII DNA ladder (Roche). Numbers designate DNA from each clone used for Southern blot analysis. Asterisk corresponds to the average position of the target DNA. (**B**) Alu-PCR analysis to validate the background integration status of aniLV episomes. Primer targets from each vector backbone (PPT-F) and Alu sequence (Alu-1) are used in combination to amplify the region between the integrated vector copy and nearby Alu fragment. M1: 100 bp DNA ladder, C1: 293T gDNA used as template (**C**) Agarose gel electrophoresis of episome-specific PCR on aniLV clones using PPT F and GFP R and the corresponding Southern blot. M1: DIG-labeled Lambda HindIII DNA ladder, M2: 1-kb DNA ladder, C1: pEpi plasmid DNA used as template, C2: 293T gDNA used as template. Number represents DNA template of each clone used for Southern blot analysis, asterisk corresponds to the average position of the target DNA. (**D**) FISH analysis on multiple metaphases of one aniLV clone (12) shows nuclear vector genomes.

In addition to detecting circular aniLV episomes via the unique PCR signature that results from LTR-recombination, we next studied genomic vector integration events by direct Southern blot analysis. Our probe was based on the pLVCG lentiviral transfer vector backbone, providing 3 kb of homology for hybridization to target genomic or episomal sequence. We extracted genomic DNA from a subset of 293T clones (3 × iLV and 15 × aniLV) performed a restriction digestion with EcoRV and hybridized for 16 h. Results show a single major DNA band at the predicted size of 6 kb in all aniLV clones studied, indicating the episomal persistence of the linearized vector in these clones after >100 rounds of replication (Figure 4A). Although background integration by niLV vectors, packaged with the integrase mutant D64V, can occur as a result of cellular recombination, no such events were obvious in the clones analyzed (8,38,39). By comparison, iLV clones included here as controls show bands of different sizes, characteristic of proviral integrants against the persistence of LTR-circles previously described by others (40). Next, to demonstrate the potential integration events of the of aniLV episomes, Alu PCR was conducted using one primer site on LV backbone and the second primer on the Alu sequence (Figure 4B). Neither genomic Southern nor Alu-directed PCR suggested DNA integration in the aniLV clones analyzed even though this might not exclude minor background integration events within clones. Additionally, we performed HIRT DNA extraction to recover extrachromosmal DNA from 293T clones. With the HIRT DNA templates we performed episome-specific ‘long-PCR’ using PPT-F + GFP-R primers to amplify a 3-kb region of the circular vector DNA containing vector backbone and transgene cassette (Figure 4C). For additional stringency, the long-PCR samples were then transferred to nylon membrane from agarose gel after electrophoresis and analyzed by DIG-labeled probe that specifically hybridized to episomal DNA. Results demonstrate the presence of specific bands at the predicted size of aniLV episomes in non-genomic DNA from 293T/aniLV clones (Figure 4C, lower panel). Further, Southern analysis of these clones and calibration against a plasmid standard curve also allowed a semiquantitative analysis of the episomal copy number. In the six clones analyzed, densitometric quantification of bands suggests the presence of up to 3–5 episomes per cell on average after >100 rounds of replication with a tendency toward lower copy numbers in qPCR assay (measured at later time points) (Supplementary Figure S4A–C).

Others have previously validated interphase and metaphase fluorescent *in situ* hybridization (FISH) analysis for visualization and to provide spatial insight into S/MAR plasmid vector episome distribution within the nucleus of target cells (25,32). We generated metaphase spreads from multiple aniLV/293T clones, using the identical probe as for Southern analysis. Our metaphase FISH studies of several aniLV clones uniformly show the LV-specific probe detection of episomal signals in cells, further confirming the persistence of aniLV in dividing cells (Figure 4C and Supplementary Figure S5).

### Anchoring niLV expression in primary murine hematopoietic progenitors *in vitro* and *in vivo*

**Figure 5.**
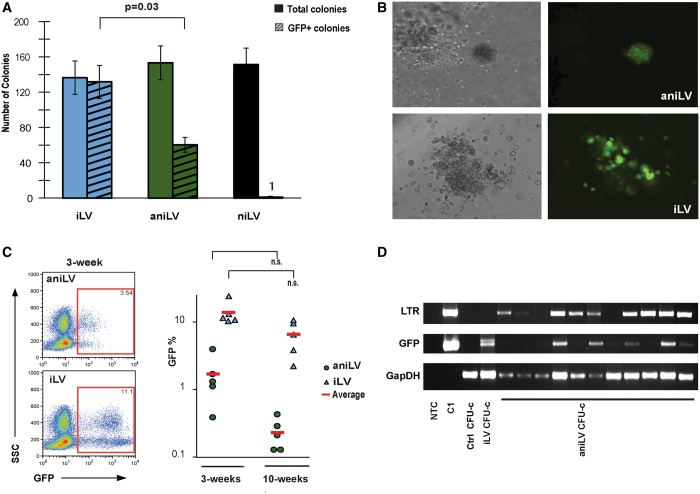
LV transduction into murine HPC *in vitro* and *in vivo*. (**A**) Colony-forming assay of LV-transduced mHPCs in cytokine-supplemented methylcellulose. GFP expression from the colonies was analyzed at 10–12 days after transduction. Total number and GFP-positive colonies were counted. (**B**) Microscopic images of GFP expression from iLV and aniLV-transduced mHPC-derived colonies. Lower GFP expression was observed in aniLV compared with iLV-transduced colonies. (**C**) GFP expression profile in aniLV-transduced mHPC-transplanted animals. Each cohort represents five animals that received aniLV-transduced mHPCs. Representative FACS analysis of whole-blood cell sample of post-transduction and transplantation of mHPCs into the mice at 3 and 10 weeks, respectively. Lower MOI was consistent with CFU assay and 293T aniLV clones compared with cells transduced with iLV. (**D**) PCR analysis to detect the presence of episomal vector DNA in the vector-transduced CFUs. LTR junction amplification using PPT-F and PBS-R primers from aniLV CFUs suggests that colonies still harbor vector episomes at detectable levels. GFP PCR as vector control and murine-specific GapDH PCR to confirm the presence of genomic DNA in positive control sample and in untransduced control CFU-c.

Gene delivery into stem cells using plasmid vectors is substantially less efficient when compared with viral vectors. For proof-of-principle that aniLV can transduce primary progenitor cell populations, we transduced lineage-depleted murine HPCs with aniLV vector and studied their progeny. Hematopoietic progenitors cultured in cytokine-supplemented semisolid media form colonies that represent the clonal outgrowth (proliferation and differentiation) of single cells. Our *in vitro* studies showed that vector exposure (aniLV or iLV) led to successful transduction of mHPCs with subsequent GFP expression in clonogenic colonies without selective pressure (Figure 5A). Results also show that vector exposure did not compromise colony formation in general, whereas aniLV transduction rates were reduced compared with iLV. Much like the data in 293T clones (Figure 2), we found that the level of GFP expression was generally decreased compared with iLV-transduced CFU-C (Figure 5B). The aggregate data in CFU-C suggested substantial potential for *in vivo* expression. Therefore, we used a murine transplantation model to track GFP expression from aniLV-transduced HPCs *in vivo*. Here, HPCs were transduced at a MOI of 10–20 overnight, washed and intravenously injected into preconditioned (750cGy) recipients. Following transplantation, we performed blood draws at 3 weeks and at 10 weeks (after hematopoietic reconstitution) to analyze the proportion of white blood cells expressing GFP by flow cytometry. Results from multiple independent transplant experiments demonstrate low, but distinct transgene expression from aniLV cohorts compared with iLV-transduced and transplanted animals. Low-level chimersim (GFP%) in the respective cohorts (iLV and aniLV) persisted at the later time point (Figure 5C). A total of four cohorts per vector (15 aniLV animals; 15 iLV animals) were studied, all showing small populations of GFP-expressing cells. Additional primary FACS files are provided for illustration in Supplementary Figure S6. The generally lower GFP expression (lower frequency and lower MFI) from aniLV episomes was consistent between *in vitro* and *in vivo* studies and corroborates the aniLV performance in dividing cell lines. To analyze the lentiviral episomal DNA in primary cells, vector-transduced single colonies (CFU-C) were picked and analyzed for LTR junctions by episome-specific PCR. Amplification of LTR junction-, murine GAPDH- and GFP sequence in DNA from single aniLV CFUs that express GFP by microscopic examination suggests the progenitor colonies harbor aniLV episomes without selection pressure (Figure 5D).

## DISCUSSION

This study investigated the long-term performance of niLVs bearing the human β-interferon S/MAR element in dividing cells. S/MAR sequences serve well-described functions during replication and by modulating gene expression (16,25,37,41). S/MAR-containing plasmids are stably maintained in dividing cells and selection for a resistance marker present within the episome has been used to achieve persistence (41–43). However, efficient delivery and cellular establishment of plasmid vectors have been a challenge (27,44). LVs on the other hand combine efficient gene delivery and active transport of lentiviral genomes to the cellular nucleus. The viral life cycle includes abortive integration events, whereby viral genomic DNAs circularize to form LTR circles and acquire an episomal status. Our studies provide proof-of-concept that the incorporation of S/MAR sequence in LVs results in long-term persistence of these niLV episomes without selection.

We mostly relied on a widely-used cell line to characterize aniLV episome performance in dividing cells and demonstrate that transduction with aniLV is followed by a period of loss of expression from most cells before stable long-term GFP expression. The frequency of 293T cells expressing GFP after this episome establishment period in bulk-purified aniLV-transduced 293T cells is maintained at <10% of the initial population. These kinetics reflect the experience by others and may indicate limited DNA tethering via the SAFA protein or loss of episome expression (despite molecular persistence) through potential epigenetic regulation, already shown for Epstein–Barr virus-derived episomes (12–14,24,25,27,45). To validate the mitotic stability of these aniLV episomes, we enriched the transduced cell population in the initial stages of culture by flow cytometric cell sorting. Next, for analysis of aniLV performance, episomal persistence and gene expression in the individual clones were tracked in detail, and we found that transgene expression was maintained through many weeks of continuous culture and repeated freeze–thaw cycles, generally echoing S/MAR episome performance after plasmid vector delivery (16,25). S/MAR sequences were previously used as insulators to regulate and optimize transgene expression in lentivectors (22,41,46,47). However, when S/MAR was cloned within the transcriptionally active region of the LV, simultaneously replacing the wPRE sequence, we found a substantial decrease in GFP expression, consistent with the experience of others (24).

However, our data in aniLV-transduced flow-cytometrically purified 293T clones and primary cells demonstrate that integration status, i.e. LTR–epsiome cassette expression, has an even greater impact on gene expression (MFI) than the presence of S/MAR sequence and reflect the general experience with non-integrating vectors, potentially indicating epigenetic regulation of established episomes (5,8,24,27,34). While precluding some applications, high levels of transgene expression are not always needed, and more physiological levels of transgene expression may increase safety (1). Apart from the mechanical (flow cytometric) enrichment of aniLV-transduced cells to analyze episome persistence and gene expression, our studies specifically avoided *in vitro* or *in vivo* selection of aniLV-transduced cells, which can boost rates of episome establishment and transgene expression (37,42,43,48,49). It is likely, that careful study of the position of the S/MAR element in the vector backbone and choice of S/MAR, other than the β-IFN element, may also help to ameliorate low expression and further optimize the performance (24,34).

Several levels of evidence support the persistence of aniLV episomes in dividing cells. As the result of abortive proviral integration of the linear vector DNA, niLV recombines through homology or non–homology-driven cellular mechanisms to form LTR circles, i.e. episomes (9,11). This provides a molecular signature distinguishing vector episomes from integrated proviral sequences. Using unique primers that span the recombined LTR, we were able to consistently amplify LTR sequence from the aniLV-transduced clones throughout the study and after many rounds of cell division without exerting selective pressure. Consistent with the predicted prevalence of circular DNA species, only one of the sequenced LTR junctions from the aniLV-transduced 293T clones showed elements of 2-LTR episomes (35). These studies confirm not only the long-term maintenance of aniLV episomes, but provide conclusive evidence for their origin from LTR recombination events. Using a ‘long-PCR’ approach combined with probe hybridization to a vector-specific probe, we further validate the presence of LTR circles bearing the GFP expression cassette in extrachromosomal DNA generated via HIRT preparation. This is complemented by genomic Southern analysis using the same hybridization probe and Alu-PCR that shows no evidence for integration in these aniLV clones. Proviral integration in iLV control clones signifies integration versus LTR circle persistence, as previously reported (40). Taken together with the sequencing of a subset of clones, the data uniformly indicate the long-term presence of episomal vector DNA in dividing cells. Finally, we relied on a validated protocol for FISH analysis and observed episomes in several clones, generally in proximity with the chromosomal arms and predominantly telomeric location, consistent with published reports (25,32). Our Southern analysis initially suggested an average of 3–5 episome copies per cell, incidentally matching prior observations on non-viral S/MAR-containing vectors. However, given the lower results of qPCR studies on these clones, our copy number estimation should not be considered conclusive, emphasizing the need to examine episome replication and segregation in mitotic cells separately. In aggregate, our data provide proof-of-principle for the aniLV persistence and corroborate the low likelihood of integration by S/MAR-based (plasmid) episomes as well as low background integration from niLV vectors (10,18,39).

The ultimate aim in developing aniLV is the delivery of genes to stem cells for long-term episomal transgene expression. Murine transplantation models provide validated preclinical models for vector development (30,50). Here we show that aniLV exposure of lineage-depleted murine hematopoietic progenitors leads to transduction and GFP expression in clonogenic colonies. This confirms reports of successful (low-level) transduction and GFP expression from S/MAR plasmids in human CD34 cells (27). Whereas aniLV/293T cell clones showed variable GFP fluorescence and evidence of silencing in some subclones, declining expression was also observed in aniLV-transduced mHPCs, revealing a consistent overall performance of aniLV episomes. When the aniLV-transduced murine HPCs were transplanted into mice, the cells readily engrafted and contributed to peripheral blood marking for up to 10 weeks, again at generally decreased GFP transgene expression levels compared with iLV.

**Figure 6.**
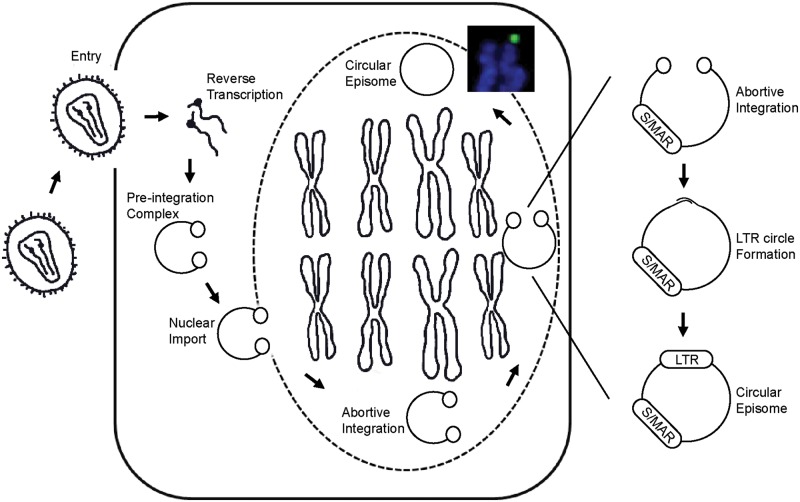
Model of aniLV delivery and nuclear retention. The vector genome undergoes reverse transcription and forms pre-integration complex after cellular entry and uncoating. The nuclear transport of the preintegration complex across the nuclear membrane delivers the vector DNA in to the host nucleus. Owing to the abortive integration, vector DNA circularizes at LTR junctions and forms S/MAR harboring episomes. Owing to S/MAR-mediated nuclear retention, episomes persist in the nucleus and attach to the nuclear matrix followed by replication and segregation during cell division.

To summarize, we report a hybrid LV that derives nuclear anchorage and mitotic replication from the S/MAR sequence. Our *in vitro* studies suggest that S/MAR niLV (i.e. aniLV) lentivector episomes undergo coordinate segregation during cell division and show long-term gene expression through successive rounds of cell division in 293T cells as well as murine HPC division and differentiation. Based on the aggregate data we propose that, once inside the nucleus, a small fraction of the aniLV episomes attain mitotic stability by localizing within specific nuclear compartments during cell division (Figure 6). Several future modifications are likely to improve long-term performance, including swapping of *cis-*regulatory elements and strategies to select for episome expression and cellular retention by replacing the GFP reporter with a therapeutic or heterologous resistance gene (48,49,51,52). Concluding, we propose further study of aniLV as a candidate vector for gene delivery to dividing stem cell populations while avoiding proviral integration and its potentially deleterious consequences.

## SUPPLEMENTARY DATA

Supplementary Data are available at NAR Online.

Supplementary Data
