# Supplementary material for: S/MAR sequence confers long-term mitotic stability on non-integrating lentiviral vector episomes without selection
Source: Nucleic Acids Res. 2014 Jan 27;42(7):e53. doi: 10.1093/nar/gku082 (PMC3985655; doi:10.1093/nar/gku082)
Supplement: Supplementary Data [file supp_42_7_e53__index.html]

S/MAR sequence confers long-term mitotic stability on non-integrating lentiviral vector episomes without selection — S/MAR sequence confers long-term mitotic stability on non-integrating lentiviral vector episomes without selection — Supplementary Data 

# S/MAR sequence confers long-term mitotic stability on non-integrating lentiviral vector episomes without selection

## Supplementary Data

files

**Files in this Data Supplement:**

- Supplementary Data - pdf file
