# Supplementary material for: S/MAR sequence confers long-term mitotic stability on non-integrating lentiviral vector episomes without selection
Source: Nucleic Acids Res. 2014 Jan 27;42(7):e53. doi: 10.1093/nar/gku082 (PMC3985655; doi:10.1093/nar/gku082)
Supplement: Supplementary Data [file supp_gku082_nar-02625-met-h-2013-File008.pdf]

Supplemental Figure 1 Vector characterization for gene expression and titer

(A) Transgene expression from Lentiviral vectors in human stromal cell (HS5) transduced at MOI 1 and analyzed the GFP expression at up to 10 days. (B) Transgene expression from Lentiviral vectors in murine cell line (NIH3T3) transduced at MOI 1 and analyzed the GFP expression up to 10 days. (C) Analysis of vector titer by Q-PCR. The concentrated vector stock was used in two different concentrations (1uL, 10uL) to transduce 293T cells and extracted the whole cellular DNA for Q-PCR.

Supplemental Figure 2. Variablity of GFP expression and episomal status in aniLV clones

(A) Cells were flow-cytometrically sorted based on GFP expression from three aniLV clones and propagated in culture separately thereafter. (B) Microscopy and FACS analysis of GFP expression from the GFP positive and -negative sorted cells from the aniLV clones after one week. Microscopic images of aniLV 17 clone maintained for one week in culture after sorting based on GFP expression. (C) PCR analysis to determine the presence of the GFP expression cassette and shows LTR episomes of aniLV irrespective of GFP expression. M: 50bp marker.

Supplemental Figure 3. Colony rescue assay to exclude plasmid contamination in LV transduced cells and LTR status in aniLV-293T cells.

A) S/MAR plasmids (pEpi and pLV-S/MAR) and non-S/MAR plasmids (pLVCG and pGFP-Vpr) were used to transfect the 293T cells. Lentiviral vectors aniLV and iLV were used to transduce the 293T cells. Whole genomic DNA was harvested at various time points after transfection and transduction and used to transform chemically competent *E.Coli* cells. Bacterial colonies grown on LB agar plates were counted. (B) Genomic DNA from aniLV clones and iLV clones were used for bacterial transformation. No significant bacterial colonies were observed from the LV delivered gDNA samples. (C) Quantification of 1-LTR and 2-LTR episomes by q-PCR. Genomic DNA of unsorted 293T cells transduced by aniLV were harvested at various time points and the DNA corresponding to 10000 cells were used as template in q-PCR quantification.

Supplemental Figure 4. iLV integration status and Quantification of aniLV episomes.

A) Southern blot analysis to identify the integrated vector copies in iLV clones. EcoR V digested DNA samples were used and the signal was detected by DIG labeled probe as shown in Fig-1A. Variable sizes of DNA signal consistent with unique proviral integration were observed in the analyzed iLV clones. (B) The S/MAR containing transfer plasmid pLV-S/MAR was used as control. A dilution series of plasmid copy numbers per lane ranges from 10^9 to 10^6 copies. Inset: GFP positive cell population of the aniLV clones by FACS analysis used for quantification. Numbers represent DNA template of each clone used for Southern Blot analysis. Asterisk corresponds to the

average position of the target DNA. (C) Analysis of episome copy number in aniLV clones by Q-PCR. The aniLV/293T clones were maintained in culture without selection pressure and extracted the whole cellular DNA at post 100 replication period. GFP standards are used to compare the copy number.

Supplemental Figure 5. FISH analysis of LV/293T clones

Metaphase spreads from LV/293T clones and non-transduced 293T cells. Genomic DNA was stained by DAPI. The FISH probe-Target DNA hybrid was detected on or near the metaphase chromosomes of aniLV and iLV transduced clones (Green). FISH studies of several aniLV clones at metaphase uniformly showed the LV specific probe detection of episomal signals in cells. No signals were detected in 293T cells and doublets were observed in some iLV clones.

Supplemental Figure 6. Lentiviral vector transduction into murine hematopoietic progenitor cells *in vivo.* Flow-cytometry dot plots with GFP expression profiles in aniLV transduced mHPC transplanted animals in 3 and 10 weeks.

S1.

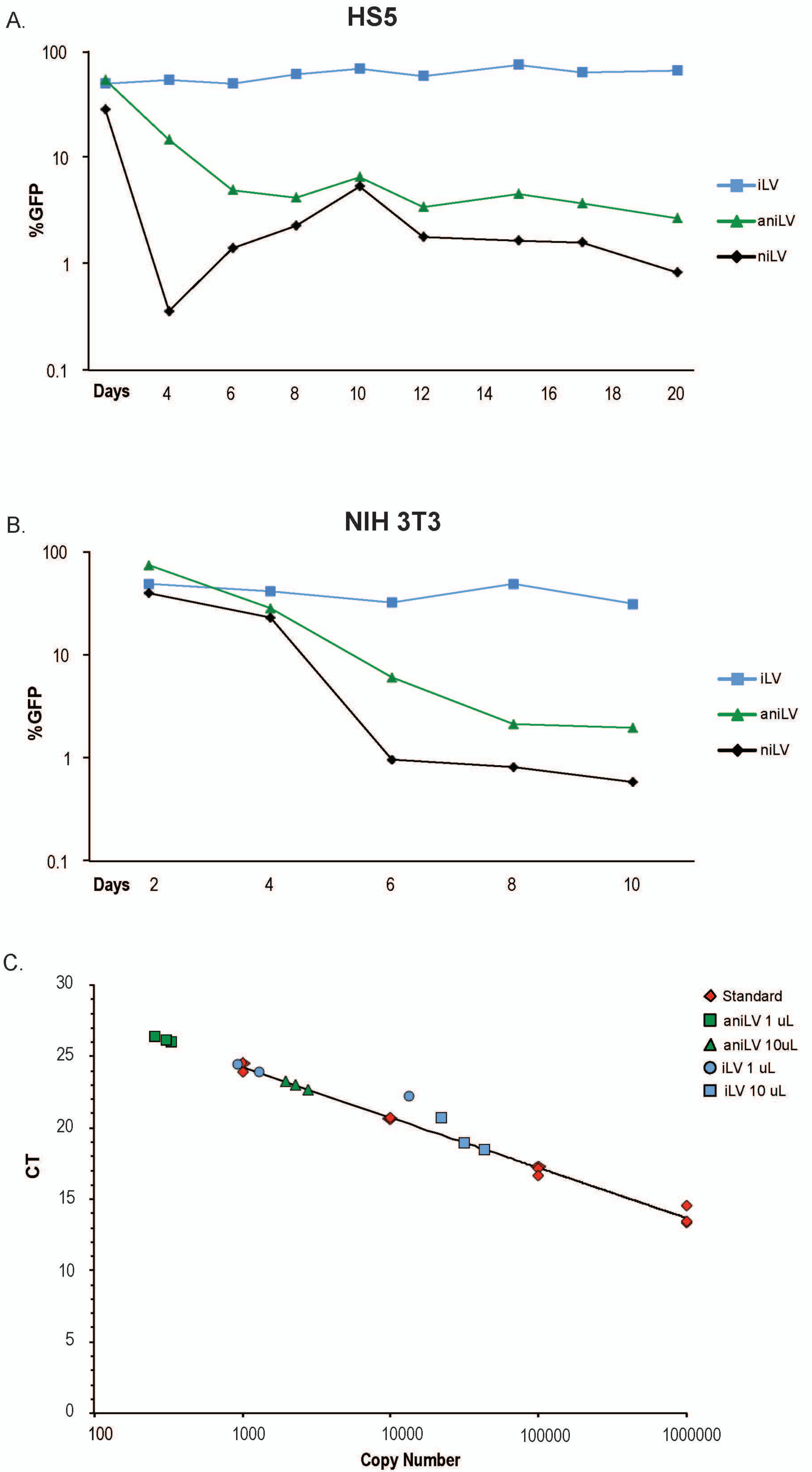
A.
HS5
100
10
1
0.1
%GFP
Days
4
6
8
10
12
14
16
18
20
iLV
aniLV
niLV
B.
NIH 3T3
100
10
1
0.1
%GFP
Days
2
4
6
8
10
iLV
aniLV
niLV
C.
30
25
20
15
10
5
0
CT
100
1000
10000
100000
1000000
Copy Number
Standard
aniLV 1 uL
aniLV 10uL
iLV 1 uL
iLV 10 uL

S2.

A.

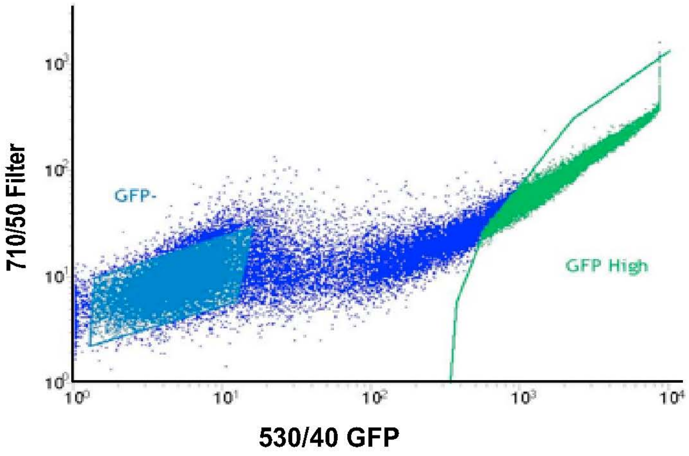

710/50 Filter
530/40 GFP
GFP-
GFP High

B.

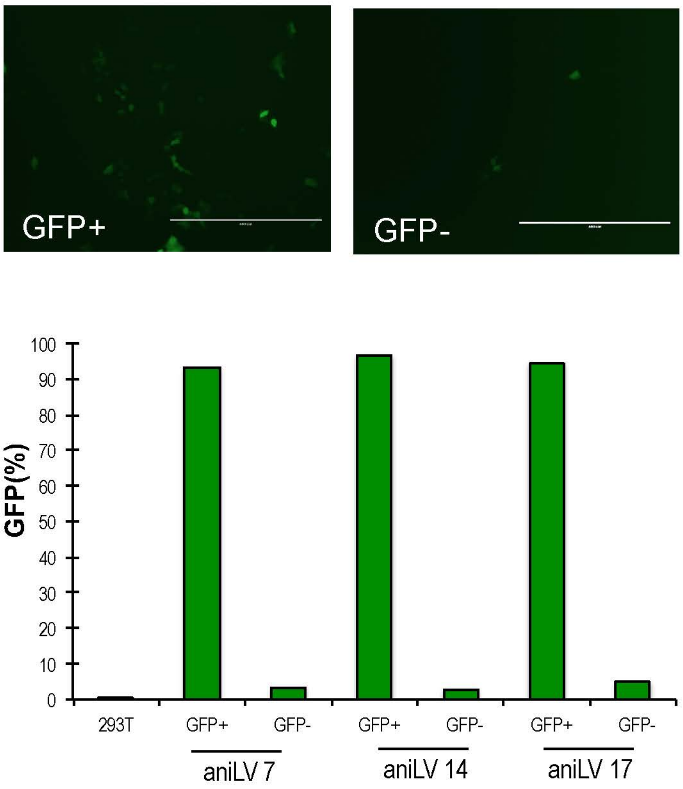

GFP+
GFP-
GFP(%)
100
90
80
70
60
50
40
30
20
10
0
293T
GFP+
GFP-
GFP+
GFP-
GFP+
GFP-
aniLV 7
aniLV 14
aniLV 17

C.

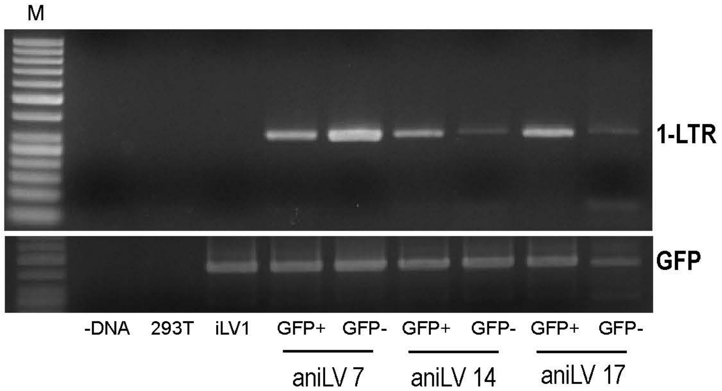

M
1-LTR
GFP
-DNA
293T
iLV1
GFP+
GFP-
GFP+
GFP-
GFP+
GFP-
aniLV 7
aniLV 14
aniLV 17

S3.

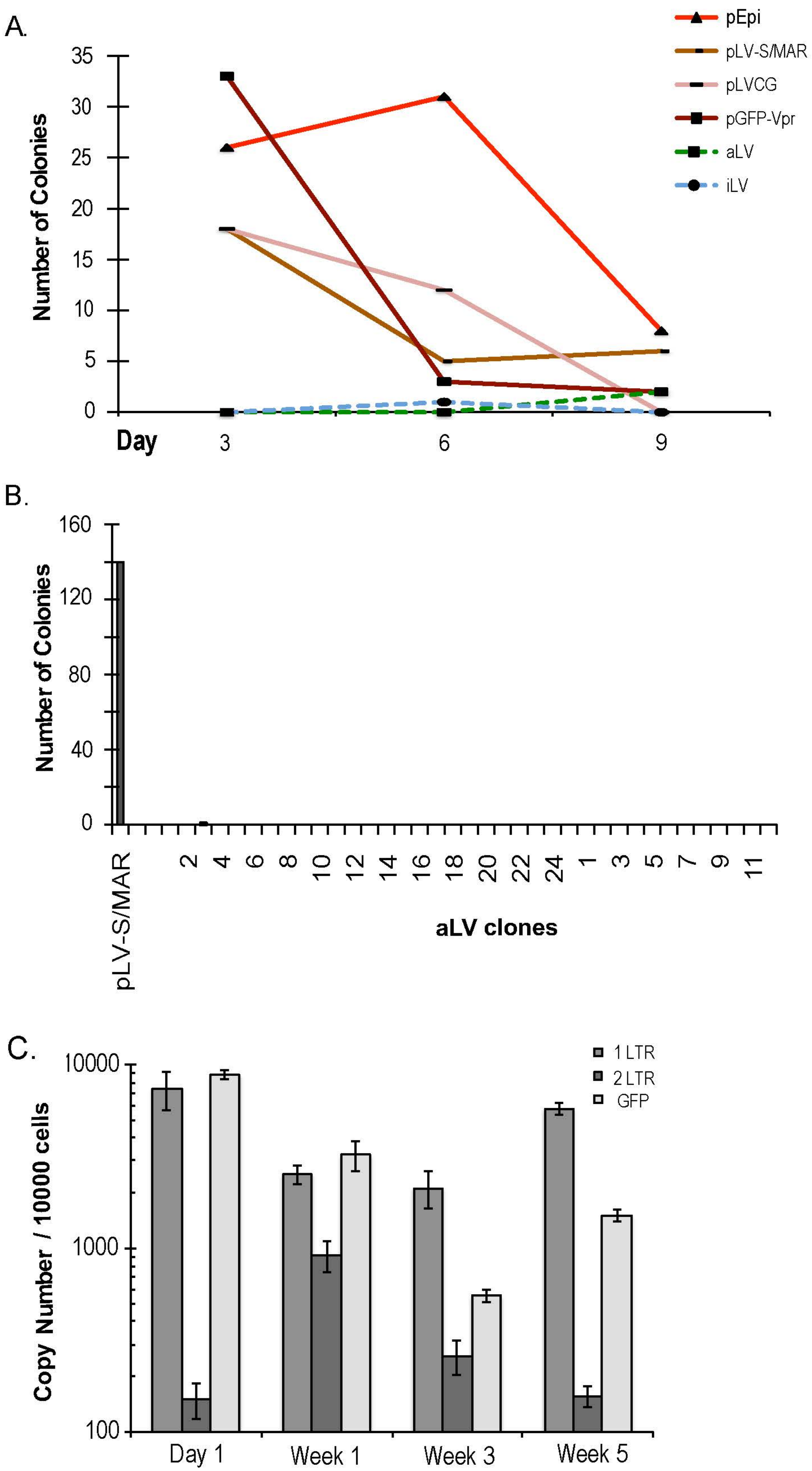
A.
pEpi
pLV-S/MAR
pLVCG
pGFP-Vpr
aLV
iLV
Number of Colonies
35
30
25
20
15
10
5
0
Day
3
6
9
B.
160
120
80
40
0
Number of Colonies
pLV-S/MAR
2
4
6
8
10
12
14
16
18
20
22
24
1
3
5
7
9
11
aLV clones
C.
1 LTR
2 LTR
GFP
10000
1000
100
Copy Number / 10000 cells
Day 1
Week 1
Week 3
Week 5

S4.

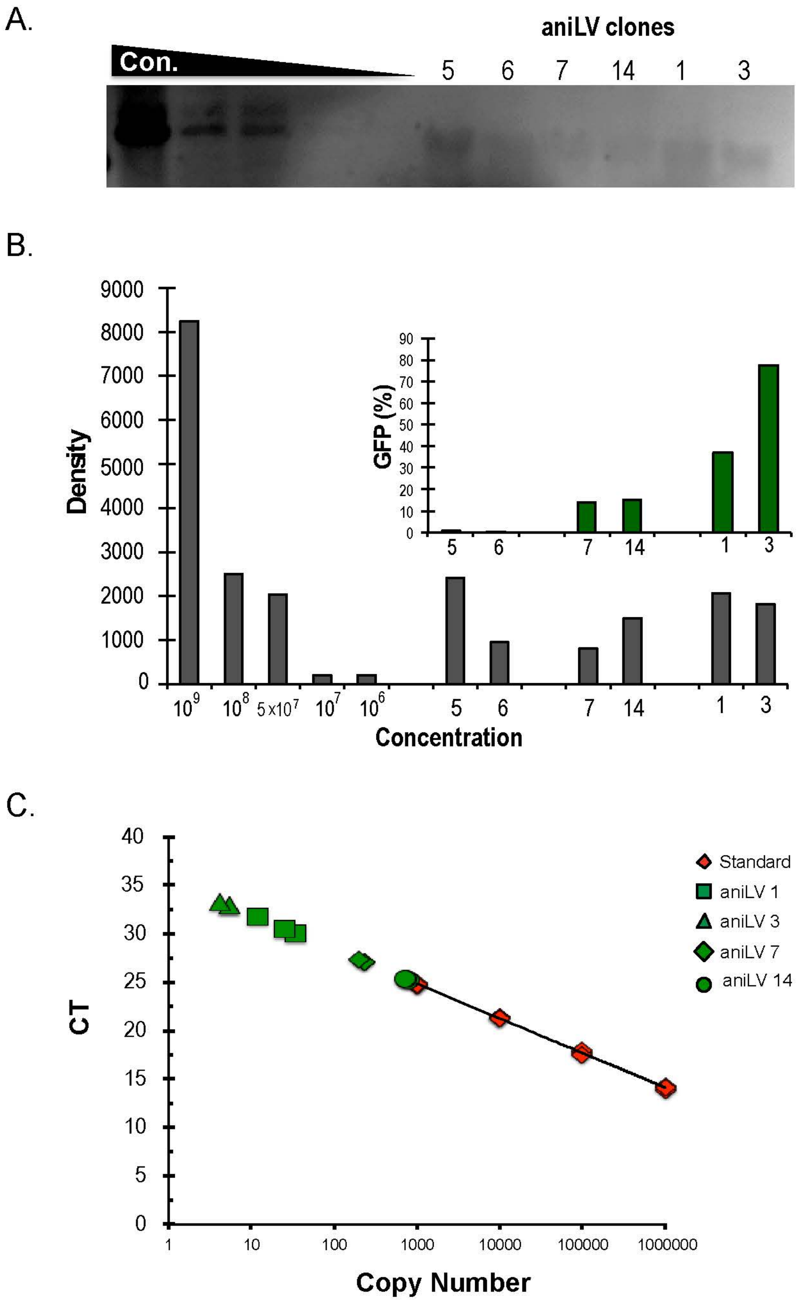
A.
aniLV clones
Con.
5
6
7
14
1
3
B.
Density
9000
8000
7000
6000
5000
4000
3000
2000
1000
0
10^9
10^8
5x10^7
10^7
10^6
5
6
7
14
1
3
Concentration
GFP (%)
90
80
70
60
50
40
30
20
10
0
5
6
7
14
1
3
C.
CT
40
35
30
25
20
15
10
5
0
1
10
100
1000
10000
100000
1000000
Copy Number
Standard
aniLV 1
aniLV 3
aniLV 7
aniLV 14

S5.

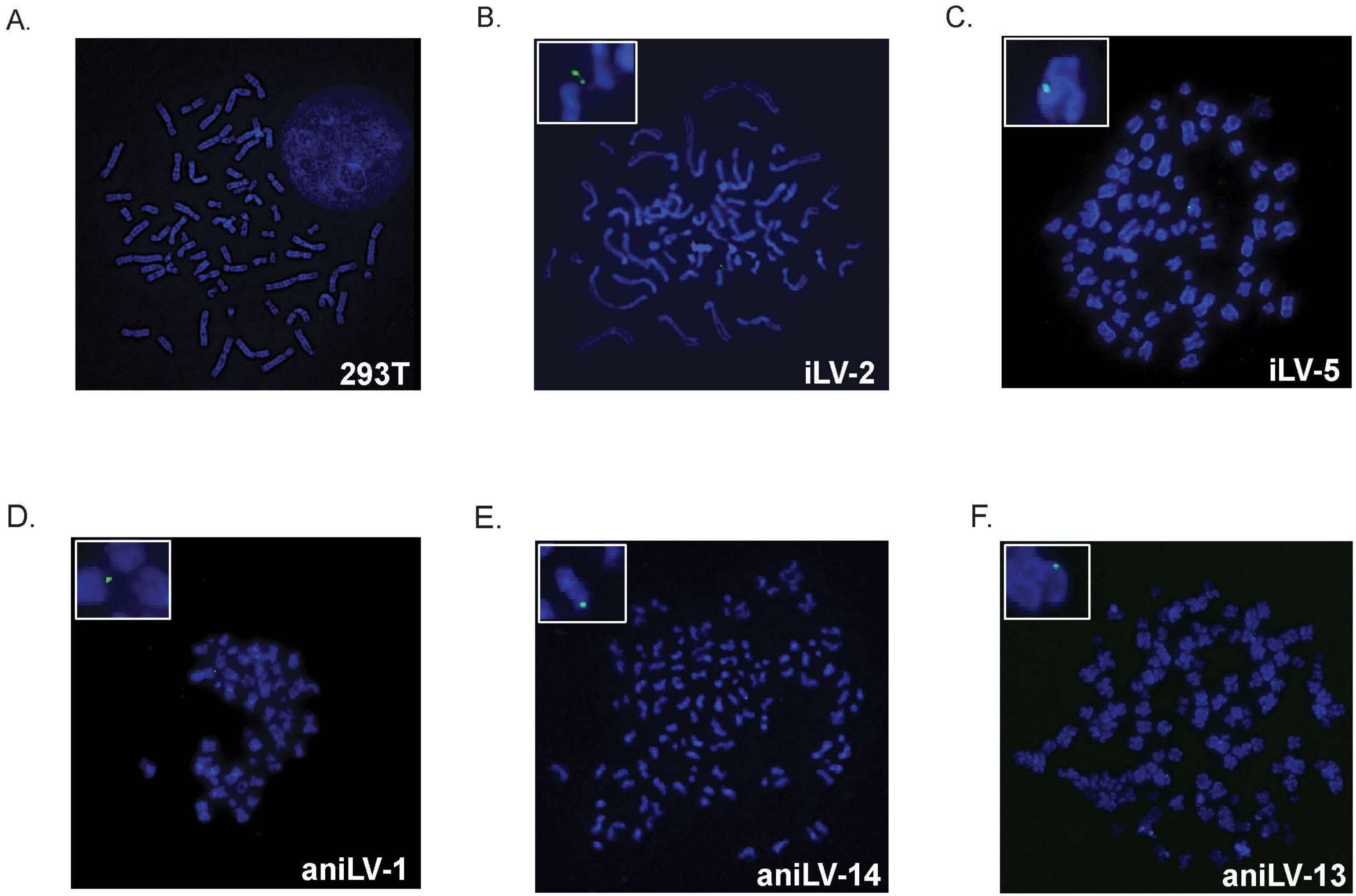

S6.

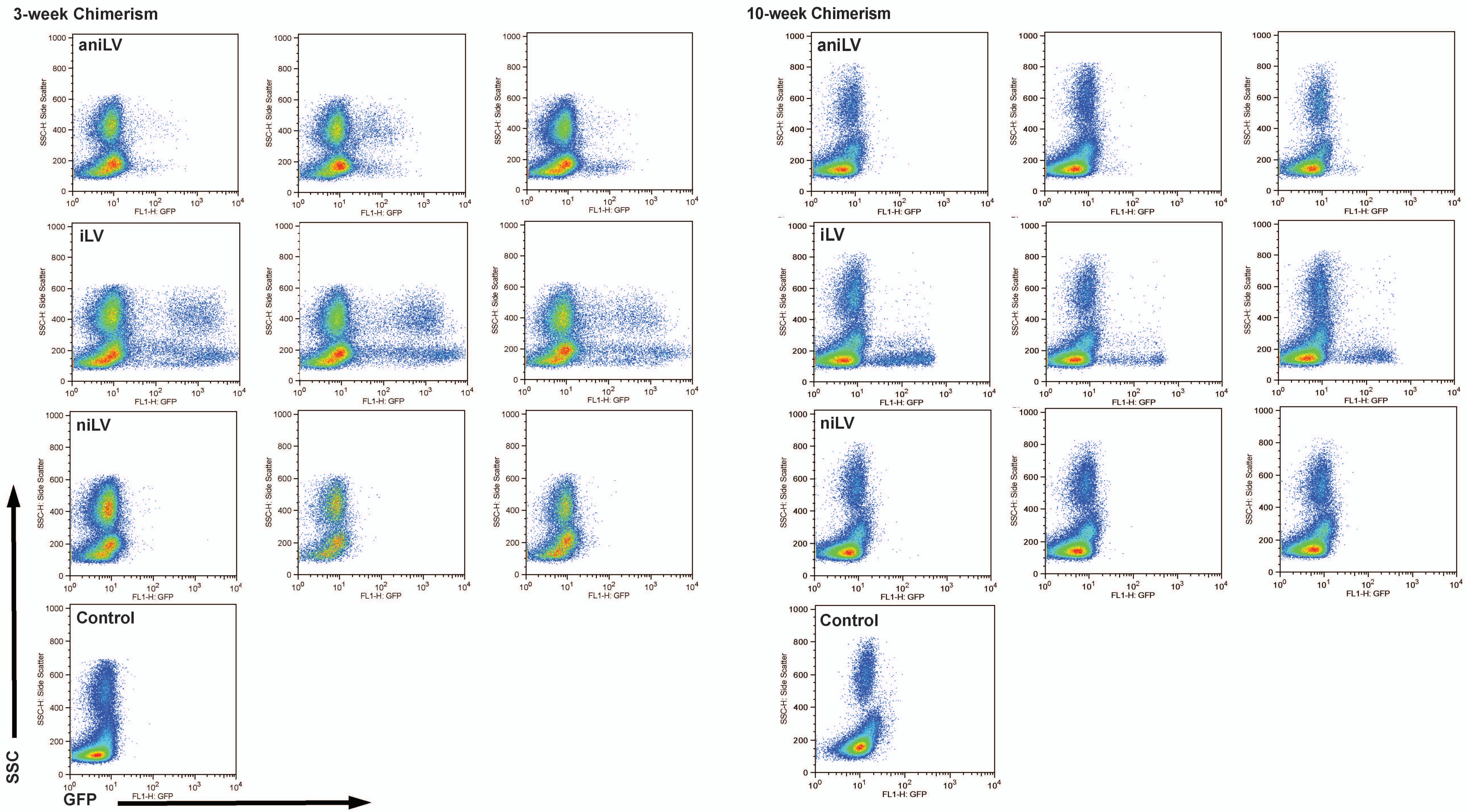
3-week Chimerism
10-week Chimerism
aniLV
iLV
niLV
Control
SSC-H: Side Scatter
FL1-H: GFP
SSC
GFP
